# Silencing DNA Polymerase β Induces Aneuploidy as a Biomarker of Poor Prognosis in Oral Squamous Cell Cancer

**DOI:** 10.3390/ijms22052402

**Published:** 2021-02-27

**Authors:** Hui-Ching Wang, Leong-Perng Chan, Chun-Chieh Wu, Shu-Jyuan Chang, Sin-Hua Moi, Chi-Wen Luo, Mei-Ren Pan

**Affiliations:** 1 Graduate Institute of Clinical Medicine, College of Medicine, Kaohsiung Medical University, Kaohsiung 807, Taiwan; joellewang66@gmail.com; 2 Department of Internal Medicine, Division of Hematology and Oncology, Kaohsiung Medical University Hospital, Kaohsiung Medical University, Kaohsiung 807, Taiwan; 3 Faculty of Medicine, College of Medicine, Kaohsiung Medical University, Kaohsiung 807, Taiwan; oleon24@yahoo.com.tw; 4 Department of Otolaryngology-Head and Neck Surgery, Kaohsiung Medical University Hospital, Kaohsiung Medical University, Kaohsiung 807, Taiwan; 5 Department of Otorhinolaryngology-Head and Neck Surgery, Kaohsiung Municipal Ta-Tung Hospital and Kaohsiung Medical University Hospital, Kaohsiung 807, Taiwan; 6 Department of Pathology, Kaohsiung Medical University Hospital, Kaohsiung Medical University, Kaohsiung 807, Taiwan; 930220@kmuh.org.tw; 7 Graduate Institute of Medicine, College of Medicine, Kaohsiung Medical University, Kaohsiung 807, Taiwan; u100800001@kmu.edu.tw; 8 Department of Chemical Engineering and Institute of Biotechnology and Chemical Engineering, I-Shou University, No.1, Sec. 1, Syuecheng Rd., Dashu District, Kaohsiung 84001, Taiwan; moi9009@isu.edu.tw; 9 Department of Surgery, Division of Breast Surgery, Kaohsiung Medical University Hospital, Kaohsiung 807, Taiwan; 1070598@kmuh.org.tw; 10 Drug Development and Value Creation Research Center, Kaohsiung Medical University, Kaohsiung 807, Taiwan

**Keywords:** oral cancer, DNA polymerase β, cell cycles, aneuploidy

## Abstract

Most patients with oral squamous cell cancer (OSCC) have a locally advanced stage at diagnosis. The treatment strategies are diverse, including surgery, radiotherapy and chemotherapy. Despite multimodality treatment, the response rate is unsatisfactory. DNA repair and genetic instability are highly associated with carcinogenesis and treatment outcomes in oral squamous cell cancer, affecting cell growth and proliferation. Therefore, focusing on DNA repair and genetic instability interactions could be a potential target for improving the outcomes of OSCC patients. DNA polymerase-β (POLB) is an important enzyme in base excision repair and contributes to gene instability, leading to tumorigenesis and cancer metastasis. The aim of our study was to confirm POLB regulates the growth of OSCC cells through modulation of cell cycle and chromosomal instability. We analyzed a tissue array from 133 OSCC patients and discovered that low POLB expression was associated with advanced tumor stage and poor overall survival. In multivariate Cox proportional hazards regression analysis, low POLB expression and advanced lymph node status were significantly associated with poor survival. By performing in vitro studies on model cell lines, we demonstrated that POLB silencing regulated cell cycles, exacerbated mitotic abnormalities and enhanced cell proliferation. After POLB depletion, OSCC cells showed chromosomal instability and aneuploidy. Thus, POLB is an important maintainer of karyotypic stability in OSCC cells.

## 1. Introduction

Worldwide, head and neck cancer (HNC) is the sixth commonest cancer, with 90% of the histological type constituted by squamous cell carcinoma [1]. In contrast to Western countries, the oral cavity is the most frequent site of the primary lesion in Asian populations, possibly due to the stimulation of carcinogens, such as areca nut, alcohol and cigarettes [2]. The characteristics of oral squamous cell carcinoma (OSCC) differ markedly from SCC of other primary sites, including the potential association with human papillomavirus infection, aggressive tumor behavior, treatment resistance and poor disease-related survival [3,4,5,6]. Surgical resection is regarded as a curative treatment for cancer of the oral cavity, although definitive concurrent chemoradiotherapy (CCRT) is the first-line treatment for inoperable locally advanced tumors. With multimodality treatment, the five-year overall survival rate is only 15–20% [7,8]. High-risk patients with extracapsular spread develop recurrence despite the administration of postoperative adjuvant CCRT [9]. Therefore, it is vital to identify prognostic biomarkers for risk stratification to guide further clinical management.

During the cancer development of OSCC, evidence indicates that malfunction of proteins promote tumorigenesis involving both enhancements of cancer cell proliferation and modulations of tumor microenvironment [10,11]. These mechanisms include persistent proliferative signals and immortality, elimination of tumor suppressive signals, maintenance of cell stemness and promotion of cancer progression and metastasis. The modulations of tumor microenvironment incorporate increasing angiogenesis and tumor-associated inflammation, reprogramming of energy metabolism and attenuation of immune attack. Additionally, genome and chromosomal instability, which produces genetic diversity, is also an indispensable part during these processes in OSCC [11]. To improve or even predict the disease outcomes, finding the key regulators which modulate these hallmarks becomes an important issue in OSCC. For example, hypoxia inducible factor-1 (HIF-1), an early regulator of tumor aggressiveness, is a key promoter of energy adaptation [12]. Its target proteins and related pathways of metabolism that are essential for the initiation and progression of OSCC. The expression of HIF-1 is also regarded as a potential prognostic biomarker in OSCC [13].

Furthermore, the OSCC is closely related to DNA damage and chromosomal instability (CIN) during tumorigenesis. Most Asian populations are exposed to carcinogens, such as areca nut, which contains arecoline. Arecoline induces DNA damage and CIN, inhibits the repair of damaged DNA and blocks the transactivation function of p53 [14,15]. In addition, several treatment strategies for oral cavity cancer lead to DNA instability and apoptosis. The pathogenesis of CIN comprises diverse gene mutations, ploidy abnormalities and chromosomal damage caused by disturbance in the checkpoint and genomic surveillance genes [16,17]. Eighty percent of human papillomavirus-negative HNC demonstrate significant chromosomal instability, and the underlying mechanisms are unclear [18,19]. Increased chromosomal instability in HNC is associated with poor outcomes, including a higher risk of lymph node metastasis and worse survival [20,21].

DNA repair genes and proteins play an important role not only in maintaining genomic integrity and avoiding DNA damage but also with metastasis and drug resistance that are associated with the high expression of the DNA repair pathway in different types of malignancy [22,23,24,25]. The DNA repair pathway mediates single-strand damage repair and double-strand break repair. POLB, known as DNA polymerase beta, is a vital enzyme of the base excision repair pathway, which is one of the major mechanisms in single-strand damage repair. When base excision repair is impaired, it results in increased genomic instability [26,27]. In different cancer cell lines and cancer patients, the expression of POLB is associated with tumorigenesis [28,29]. The survival of OSCC in African Americans is highly associated with POLB as indicated through integrative genomic analysis [30]. Therefore, there is a strong connection between POLB and the occurrence of oral cavity cancer. However, the role of POLB in CIN remains elusive.

In previous studies, the gene mutation and protein expression of POLB were associated with tumor metastasis in several cancer types [31,32]. According to ONCOMINE analyses for different types of malignancy, higher expression levels of DNA repair proteins are observed in various cancers, including HNC [33]. Nevertheless, the precise role of POLB in oral cavity cancer is still unknown. In this study, we aimed to investigated whether POLB interferes with the cell cycle and genomic integrity of OSCC and then modulates the cancer cell growth.

## 2. Results

### 2.1. Correlations between POLB and Clinicopathological Parameters

**Figure 1:**
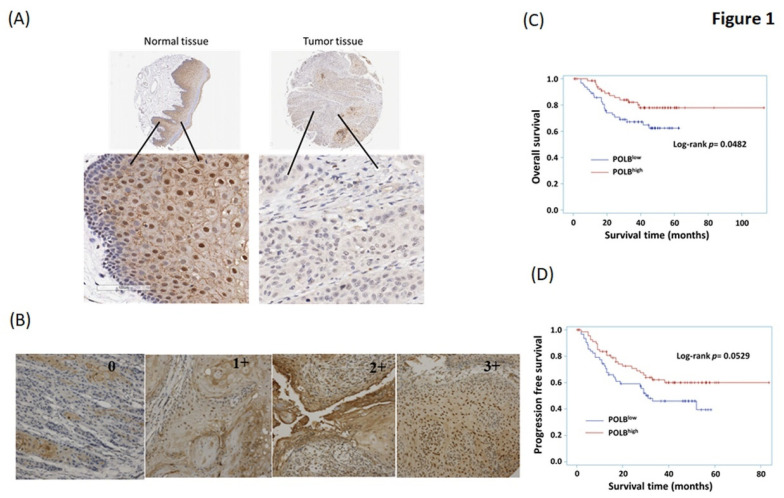
Representative immunohistochemistry staining results of POLB in tumor cells of OSCC patients, evaluation of POLB expression as a favorable prognostic marker in OSCC patients, and comparison of POLB protein expression between normal and OSCC cells in tissue array. (**A**) IHC representative images of POLB in OSCC cancer tissue sections are shown. (**B**) Immunoreactivity of POLB was classified as negative or positive (1+, 2+ and 3+) according to staining observed in the cell nucleus; magnification, 200×. (**C**) The Kaplan–Meier curves of progression-free survival for our OSCC patients. (**D**) The Kaplan–Meier curves of overall survival for our OSCC patients.

To determine whether POLB is an important prognostic marker in OSCC patients, we used immunohistochemical staining to explore the relationships between POLB and clinicopathological parameters in biopsy specimens from 133 patients with oral cavity cancer. We firstly compared the basal expression of POLB in normal oral epithelial cells and tumor part. We found that the POLB expression in cancer cells from OSCC patients was lower than that in normal oral epithelial cells (Figure 1A). Next, we collected data on the clinical parameters, including stage, lymph node and distant metastases, histological grade, progression-free survival and overall survival. The basic clinical characteristics of the study participants are listed in Table 1. The mean age was 55.05 ± 10.29 years, 94% of patients were male, 61.7% of cancers were in the buccal area and most of the patients had a history of consumption of tobacco (84.2%), alcohol (63.9%) and betel nut (76.7%). The median survival of the OSCC patients was 37.22 ± 19.17 months (0.90–113.00 months). Figure 1B shows 0, 1+, 2+ and 3+ expression of POLB in these OSCC patients. Table 2 indicates that POLB expression was significantly associated with tumor stage and survival in OSCC patients (*p* = 0.0176 and *p* = 0.0325, respectively).

### 2.2. High Level of POLB Is Positive Correlated with Poor Outcome of OSCC

We explored the relevance of POLB expression with regard to clinical outcomes in OSCC patients. We retrospectively analyzed the prognostic significance of baseline POLB expression from oral cavity samples on the overall survival and progression-free survival of 133 OSCC patients. The results of Kaplan–Meier survival analysis showed a statistically significant inverse correlation between POLB levels and overall survival (*p* = 0.048, Figure 1C). Patients with high POLB expression had significantly longer overall survival than patients with low POLB expression. However, there was only a trend of a correlation between POLB expression and progression-free survival (*p* = 0.053, Figure 1D).

We further investigated the univariate and multivariate results of Cox regression model proportional hazards regression analysis (Table 3). In the univariate Cox regression model, overall survival was only significantly associated with a higher nodal status (*p* < 0.001). Further analysis using a multivariate Cox regression model, which controlled for the effect of other clinical parameters, indicated that patients with high POLB expression were 0.475 times more likely to die of OSCC than those with low POLB expression (95% confidence interval (CI) of hazard ratio (HR) = 0.233–0.965), which also suggested that POLB expression had a protective effect on overall survival (*p* = 0.04). Together, these data suggest that POLB is a biomarker of OSCC and provides a rationale for targeting POLB in OSCC. In addition, higher nodal status (N1–N3) was significantly associated with poor overall survival in multivariate Cox regression analysis (HR = 3.441, 95% CI = 1.710–6.927).

### 2.3. Loss of POLB Increases Cell Proliferation of OSCC Cancer

**Figure 2:**
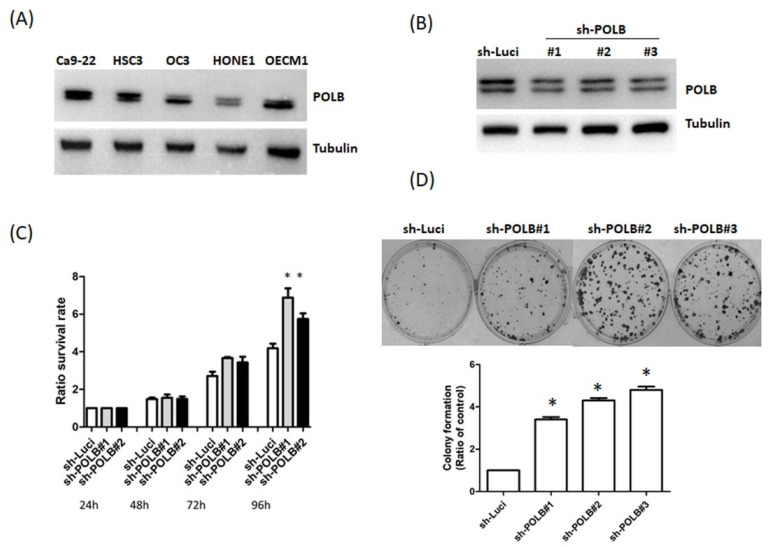
POLB knockdown triggers cell proliferation of OSCC cells. (**A**) Expression of POLB in five OSCC cell lines (Ca9-22, HSC3, OC3, HONE1 and OECM1). Cell lysates were immunoblotted by anti-POLB. Tubulin was used as a loading control. Experiments were performed independently at least three times. (**B**) The level of POLB protein was determined using Western blot analysis in stably POLB-depleted Ca9-22 cells. (**C**) MTT assays were performed for 24, 48, 72 and 96 h in parental and POLB-depleted cells. Values represent the mean ± SD from at least three independent experiments. Statistically significant data are indicated by * for significance at *p* < 0.05. (**D**) Clonogenic assay were performed in 5 × 10^2^ parental Ca9-22 and POLB silencing (shPOLB) Ca9-22 cells. Quantitative analyses of clonogenic assay are shown in the right panel.

To verify the role of POLB in OSCC, we first compared the basal expression of POLB in four OSCC cell lines (Ca9-22, HSC3, OC3 and OECM1). Ca9-22 displayed a higher level of POLB expression than other cell lines (Figure 2A). As shown in Table 2, there was a positive correlation between the levels of POLB expression and tumor stage. To confirm the role of POLB in cell proliferation, we established POLB stable knockdown cells (Figure 2B). As shown in Figure 2C, cell proliferation was more prominent in POLB-silenced Ca9-22 cells than in parental Ca9-22 cells. To determine whether POLB silencing promotes a malignant phenotype, we performed colony-formation assays. The number of colonies formed by POLB-depleted cells was higher than that formed by parental cells (Figure 2D). These findings support an important role of POLB in cell proliferation. 

### 2.4. Depletion of POLB Leads to Cell-Cycle Dysregulation and Aneuploidy Formation

**Figure 3:**
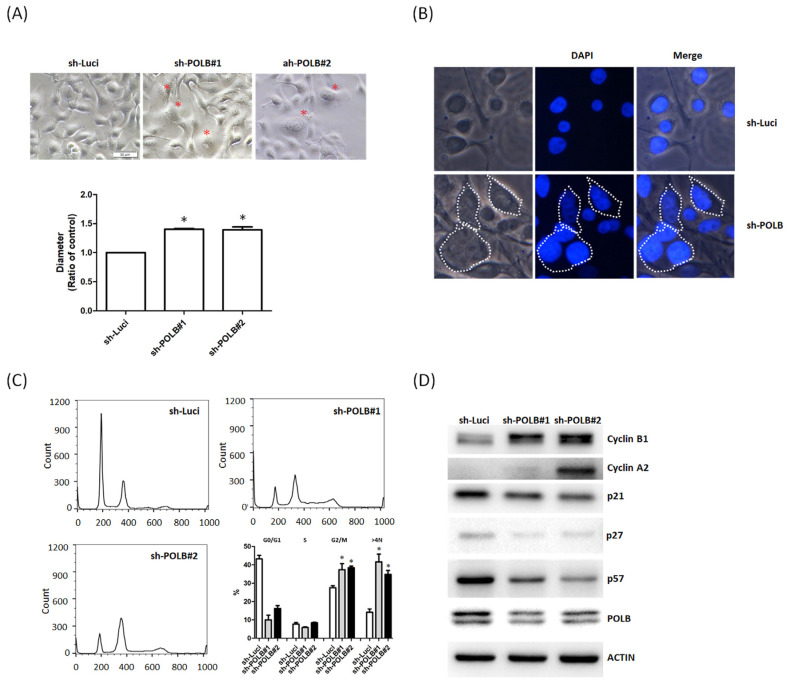
Depletion of POLB induces aneuploid cells in Ca9-22 cells. (**A**) Morphology of Ca9-22 and POLB-depleted Ca9-22 cells were visualized under a light microscope. Candidate binucleated and multinucleated cells are indicated with red stars. Quantitative analyses of nuclear size are shown in the right panel. (**B**) Cell nuclei of Ca9-22 and POLB-depleted Ca9-22 cells were stained with DAPI solution (blue color). The fluorescent image was observed under a fluorescence microscope. (**C**) Next, cells were stained with propidium iodide (PI) followed by flow cytometry analysis to obtain cell cycle distribution of Ca9-22 and POLB-depleted Ca9-22 cells. Data are representative of three independent experiments. N, haploid number. (**D**) Levels of indicated proteins were analyzed by Western blotting in Ca9-22 and POLB-depleted Ca9-22 cells. Anti-actin was used as a loading control. Statistically significant data are indicated by * for significance at *p* < 0.05.

Notably, the morphology of Ca9-22 cells changed, and they transformed into bizarre-shaped cells with prominent nuclear size and into binucleated and multinucleated cells, as shown in the fluorescence microscopy images of POLB-depleted cells (Figure 3A,B). The size of Ca9-22 cells with knockdown of POLB was larger than that of Ca9-22 cells. These results suggest that POLB may be involved in aneuploidy-associated OSCC cells. To confirm the cell cycle block or abnormal accumulation of tetraploid/near-tetraploid cells, we performed further flow cytometric analysis by using propidium iodide. The shutdown of POLB in Ca9-22 cells resulted in a significant increase in the percentage of polyploid and aneuploid cells with >4 N compared with parental Ca9-22 cells (Figure 3C). It has been known that Cdk1-cyclin B1 kinase activity is required for meiotic maturation [34]. To explore the morphological differences between cells with or without POLB, we examined the expression of the associated cyclin and cyclin-dependent kinase (CDK) inhibitors using immunoblot assays. After the shutdown of POLB expression, the levels of CDK inhibitors, including p21, p27 and p57, were decreased. Notably, the levels of cyclins, such as cyclin A2 and cyclin B1, increased after the depletion of POLB (Figure 3D). The results suggest that POLB silencing promoted polyploidy and aneuploidy through dysregulation of cyclin B and cyclin A2. The abovementioned findings suggest the importance of regulation with POLB for the maintenance of karyotypic stability in OSCC.

## 3. Discussion

More than 80% of head and neck malignancies are classified as OSCC [35]. Despite advances in therapeutic approaches, the prognosis of OSCC has not improved significantly during the past decades: 40% of OSCC patients die from locoregional disease, while 24% of patients develop metastases to distant sites [36]. Identifying novel biomarkers to predict survival and prognosis is urgently needed for the management of OSCC patients.

In previous reports, prognostic biomarkers for head and neck cancer and some relevant studies have been published. In Taiwanese OSCC patients, upregulation of *APOBEC3A* expression is correlated with better overall survival in patients carrying *APOBEC3B*-deletion alleles [37]. From the GSE 25099 of Gene Expression Omnibus (GEO) and The Cancer Genome Atlas (TCGA) databases, a set of potential prognostic signatures associated with overall survival in OSCC patients was identified, including *PLAU*, *CLDN8* and *CDKN2A* [38]. In the meta-analysis for OSCC, CD163+ M2 macrophages and CD57+ natural killer cells were the most promising predictors of survival among the tissue-infiltrating immune cells [39]. However, more studies are still warranted for improving outcomes and prognosis in OSCC patients.

POLB, belonging to the family of X DNA polymerases (pol X), is the main enzyme involved in base excision repair of DNA damage. Previous studies found a higher incidence of head and neck squamous cell carcinoma, especially oral cavity cancer and laryngeal cancer, in African Americans. An integrative genomics analysis in African Americans identified the association of POLB and genetic ancestry with survival disparity in HNSCC [30]. High levels of POLB are closely associated with tumor metastasis and poor prognosis in other malignancies, such as esophageal cancer and gastric cancer [31,32]. Previous studies have demonstrated that POLB regulates genetic instability of solid tumors, including frequent gains and losses of whole chromosomes, to interfere with spindle assembly for chromosomal segregation [29,40]. However, the role of POLB in oral cancer remains unclear. The mechanisms involving POLB in the modification of the mitotic checkpoint deficit and the cell cycle remain elusive. These findings suggest that the POLB expression may play an important role in the clinical outcomes and survival of OSCC patients. In our analysis, however, upregulation of POLB was associated with favorable outcomes for the 133 OSCC patients included in this study.

Furthermore, differences in POLB expression are demonstrated in the morphology and cell proliferation patterns in this in vitro study. Ca9-22 cells, with higher POLB expression, showed less aggressive morphological patterns compared with Ca9-22 cells with knocking down of POLB. Given that the functional properties and morphology of oral cancer cells are affected by the presence of POLB, we further examined whether these changes could be correlated to cell-cycle regulation and chromosomal instability. As expected, the existence of POLB influences the cell cycle and alters the expression of cyclin-dependent kinases and the associated inhibitors. To clarify that this effect is mainly due to numerical chromosomal changes or activation of the checkpoint in diploid cells, we performed flow cytometric analysis to determine the cell-cycle distribution of OSCC cells with or without POLB. In OSCC cells, depletion of POLB increased the proportion of polyploid and aneuploid cells with DNA content of 8N and >4N, revealing that the loss of POLB disrupts the normal cell-cycle progression, leading to aneuploidy and genetic instability in OSCC.

Previous reports indicate that POLB plays a pivotal role in genetic and chromosomal instability [41,42,43]. Several studies have demonstrated that POLB overexpression triggers an alternative DNA repair pathway that leads to genetic instability in mammalian cells [44]. However, in this study, POLB had a positive and protective impact on OSCC patients, which is different from the finding of previous studies of other cancer types [45,46]. Chromosomal instability is involved in both DNA damage and double strand break, which means regulation of POLB may be a potential strategy of combination treatment, such as DNA damage agents, cell cycle regulators and even immunotherapy. However, there are some limitations in our study, including uneven distribution of subgroups and lack of functional assays and in vivo experiments. Besides, the results in Table 3 demonstrate patients with higher grade of OSCC had relatively better survival in the univariate Cox regression model, which may relate to the case number was less in the grade 3 group (N = 2). Similarly, the HR of areca nuts was insignificant in the univariate Cox regression model but significant in the multivariate Cox regression model. We supposed there may be some interactions between areca nuts and POLB. However, further related investigations were warranted.

In conclusion, we demonstrated that low expression of POLB was associated with advanced tumor stage and worse overall survival in patients with oral cancer. POLB expression is correlated with early tumor stage and better survival, and it is an independent biomarker for predicting favorable prognosis in OSCC. Furthermore, we identified an important role of POLB in the regulation of the cell cycle. In vitro experiments demonstrated that silencing of POLB could induce chromosomal instability and numerical alterations. In brief, we confirmed POLB guarded the cell cycle and maintained the integrity of chromosomes which may provide a modifiable target for future therapeutic strategies in OSCC patients. In the future, POLB can be used in accordance to other biomarkers of different pathways to further create “a biomarker panel” for an individual prognostic calculation in OSCC patients.

## 4. Material and Methods

### 4.1. Specimens

Tissue samples of 163 representative oral cavity cancer patients treated from 2012 to 2014 were obtained from the Department of Pathology, Kaohsiung Medical University Hospital, Kaohsiung (KMUH), Taiwan. Three tissue cores (2 mm in diameter) were obtained from each paraffin block, from which three cores of cancerous tissue were cut longitudinally. The tissue specimens were set in blocks, using a fine steel needle, to produce tissue microarrays. The blocks were fixed with formalin, embedded in paraffin and stained with hematoxylin and eosin. Clinicopathological data were collected from the cancer registry and medical records of the KMUH. The use of human specimens and clinical data for this study was approved by the institutional review board and ethics committee of Kaohsiung Medical University Hospital and the institutional review board of Academia Sinica (KMUHIRB-E(I)-20170034). The data were analyzed anonymously and, therefore, the approval committee waived the requirement for informed consent. All procedures were performed in accordance with the guidelines and regulations.

### 4.2. Immunohistochemical Staining

Immunohistochemical staining was performed as previously described [47]. Briefly, human tumor samples were cut into 4-mm-thick sections and deparaffined in xylene as previously described [29]. Endogenous peroxidase activity was blocked by 3% hydrogen peroxide (Sigma, St. Louis, MO, USA) for 6–8 min. The tissues were then incubated with POLB primary antibodies for 1 h at room temperature and subsequently rinsed three times with phosphate-buffered saline according to the manufacturer’s instructions. After incubation for 30 min at 25 °C with secondary antibodies and a horseradish peroxidase/Fab polymer conjugate (EnVision™ Detection systems Peroxidase/DAB, Rabbit/Mouse [K5007 HRP; DaKo; Agilent Technologies, Inc., Santa Clara, CA, USA]), the tissues were rinsed three times with phosphate-buffered saline. Finally, the chromogen was developed with 3,3-diamino-benzidine tetrahydrochloride as the substrate, and the sections were counterstained with hematoxylin for 90 s and examined under a microscope. Negative controls were established by replacing the primary antibody with non-immune serum.

### 4.3. Scoring

The scoring criteria used for different patients were the same as in a previous study [48], and were divided into two parts: the intensity of the signal (0, 1+, 2+ and 3+) and the proportion of positive cells (0, <10%; 1, 10–25%; 2, 25–50%; 3, >50%) [49]. The staining index was calculated as the product of signal intensity and the proportion of positive cells. A score of ≤4 and ≥6 was defined as indicating low and high expression, respectively. The staining intensity in the cancerous tissue was examined by two pathologists independently.

### 4.4. Cell Culture

The dysplastic oral leukoplakia cell line (DOK) and oral cavity cancer cell lines (Ca9-22, HSC3, OC3 and OECM1; ATCC, Manassas, VA, USA) were used in this study. Cells were cultured in Dulbecco’s modified Eagle’s medium (DMEM) and DMEM/F12 medium with 10% fetal bovine serum (Hyclone Laboratories Inc., South Logan, UT, USA) and antibiotics, at 37 °C in 5% CO_2_.

### 4.5. Antibodies

Antibodies against actin (GTX11003) and Tubulin (GTX628802) were purchased from GeneTex International (Irvine, CA, USA). Cyclin B1 (#4138), cyclin A2 (#4656) and p21 (#2947) antibodies were purchased from Cell Signaling Technology (Danvers, MA, USA). p27 (ab32034) and p57(ab75974) antibodies was purchased from Abcam (Cambridge, MA, USA). POLB antibody (AP50642) was from Abgent (San Diego, CA, USA).

### 4.6. Western Blotting

Extraction and immunoblotting of proteins were performed as previously described [50]. Cells were lysed in protein lysis buffer (M-PERTM mammalian protein extraction buffer; Thermo Scientific, Rockford, IL, USA), and cellular debris was removed by centrifugation at 13,000 rpm. Target proteins were collected; their expressions were quantified; they were separated by sodium dodecyl sulfate–polyacrylamide gel electrophoresis (SDS-PAGE) and transferred to a nitrocellulose membrane; and, finally, they were immunoblotted using the indicated antibodies.

### 4.7. POLB Short Interfering RNA Transfection

For knockdown Pol β, short hairpin (sh)RNA against Pol β were purchased from the National RNAi Core Facility (Academia Sinica, Taipei City, Taiwan). The following oligonucleotide was used: human Pol β shRNA: POLB:#1-CCTGTCAAAGGGTGAGACAAA; POLB:#2-CCAGCTTCACTTCAGAATCAA; POLB:#3-GCTAAGAAATTGCCTGGAGTA; shRNA against luciferase (sequence: GCGGTTGCCAAGAGGTTCCAT). Cells were transfected with 100 nM non-targeting and specific siRNA using Lipofectamine 2000 and Opti-MEM medium according to the standard protocols specified by Invitrogen (Carlsbad, CA, USA).

### 4.8. Cell Viability Test

The 3-(4, 5-dimethylthiazol-2-yl)-2, 5-diphenyltetrazolium bromide (MTT) assay was used to assess cell viability [51]. In total, 3000 cells were seeded in 96-well plates. Cells were treated with the indicated drugs for 72 h. After inoculation, cells were incubated with 0.5 mg/mL of MTT at 37 °C for 2 h. The medium was replaced by 100 μL DMSO per well to dissolve the precipitates. Colorimetric analysis was performed using a 96-well microplate reader (BioTek Instruments, SYNERGY HTX, Vermont, USA) at a wavelength of 570 nm.

### 4.9. Flow Cytometry for Cell-Cycle Analysis

Ca9-22 cells were transfected with sh-POLB, harvested via trypsinization and fixed with 70% ice-cold ethanol overnight at −20 °C. On the following day, the cell pellet was resuspended in propidium iodide-staining buffer (50 μL/mL PI, RNAse A, Beckman Coulter, Brea, CA, USA) and incubated for 15 min at 37 °C for further cell-cycle analysis. Cell-cycle distribution was analyzed via FACS Calibur (BD Biosciences, San Diego, CA, USA) using ModFit LT v3.3 software.

### 4.10. Statistical Analysis

The expression of POLB in oral cavity cancer determined by immunohistochemical staining was evaluated by chi-square tests. To assess the value of POLB for oral cavity cancer prognosis, we performed the Kaplan–Meier method for survival curves. A Cox proportional hazards model was used to estimate univariate comparisons of overall survival and progression-free survival with clinicopathologic variables. Two-tailed Student’s *t*-test was used to evaluate differences between the groups. *p* < 0.05 was defined as statistically significant. All statistical analyses were performed using SPSS 22.0 software (IBM Corp., Armonk, NY, USA).

## Figures and Tables

**Table 1:** Characteristics of patients with oral cavity squamous cell carcinoma.

Characteristics	All (*n* = 133)
Age, years	55.05 ± 10.29
Sex, No. (%)	
Male	125 (93.98)
Female	8 (6.02)
Tumor site, No. (%)	
Buccal	82 (61.65)
Non-buccal	51 (38.35)
Alcohol, No. (%)	
Yes	85 (63.91)
No	48 (36.09)
Areca nuts, No. (%)	
Yes	102 (76.69)
No	31 (23.31)
Smoking, No. (%)	
Yes	112 (84.21)
No	21 (15.79)
Lymph node metastases, No. (%)	
Yes	33 (24.81)
No	100 (75.19)
Grade, No. (%)	
1	59 (44.70)
2	71 (53.79)
3	2 (1.52)
Stage, No. (%)	
I	59 (44.70)
II	23 (17.42)
III	15 (11.36)
IV	35 (26.52)

**Table 2:** Relationship between POLB expression and clinicopathological characteristics of oral cancer patients (*n* = 133). Statistical analysis was performed using the Chi-squared test.

Parameters	*n*	POLB Expression, *n* (%)		*p*-Value
		Low	High	
Total	133	63 (47.37)	70 (52.63)	
Age				0.1893
≤40 yrs	5	4 (6.35)	1 (1.43)	
>40 yrs	128	59 (93.65)	69 (98.57)	
Gender				0.0068 *
female	8	0 (0.00)	8 (11.43)	
male	125	63 (100.00)	62 (88.57)	
Tumor size				0.0961
≤2.0 cm	65	26 (41.27)	39 (55.71)	
>2.0 cm	68	37 (58.73)	31 (44.29)	
Grade				0.6009
I	59	26 (41.94)	33 (47.14)	
II/III	73	36 (58.06)	37 (52.86)	
Tumor stage				0.0176 *
T1/T2	101	42 (66.67)	59 (84.29)	
T3/T4	32	21 (33.33)	11 (15.71)	
Nodal stage				0.5822
N0	100	46 (73.02)	54 (77.14)	
N1/N2/N3	33	17 (26.98)	16 (22.86)	
Metastatic stage				0.3410
M0	132	63 (100.00)	69 (98.57)	
M1	1	0 (0.00)	1 (1.43)	
Tumor metastasis				0.1907
Absent	123	56 (88.89)	67 (95.1)	
Present	10	7 (11.11)	3 (4.29)	
Tumor recurrent				0.1045
Absent	82	34 (54.84)	48 (68.57)	
Present	50	28 (45.16)	22 (31.43)	
Survival status				0.0325 *
Survival	98	41 (65.08)	57 (81.43)	
Death	35	22 (34.92)	13 (18.57)	
Alcohol				0.9242
N	48	23 (36.51)	25 (35.71)	
Y	85	40 (63.49)	45 (64.29)	
Areca nuts				0.8968
N	31	15 (23.81)	16 (22.86)	
Y	102	48 (76.19)	54 (77.14)	
Smoking				0.146
N	21	13 (20.63)	8 (11.43)	
Y	112	50 (79.37)	62 (88.57)	

* Statistically significant (*p* < 0.05).

**Table 3:** Univariate and multivariate logistic analysis of clinicopathological independent prognostic factors for survival of oral cancer patients (*n* = 133). Statistical analysis was performed using Cox multivariate analysis.

Factors	Univariate		Multivariate	
	HR (95% CI)	*p*-Value	HR (95% CI)	*p*-Value
POLB expression		0.0688		0.0360 *
Low	1.0		1.0	
High	0.526 (0.263–1.051)		0.464 (0.226–0.951)	
Tumor Size		0.0055 *		0.1436
≤2.0 cm	1.0		1.0	
>2.0 cm	2.945 (1.373–6.315)		1.846 (0.812–4.198)	
Grade		0.0081 *		0.1823
I	1.0		1.0	
II/III	0.356 (0.166–0.765)		0.578 (0.259–1.293)	
Nodal stage		0.0002 *		0.0065 *
N0	1.0		1.0	
N1/N2/N3	3.534 (1.799–6.942)		2.777 (1.330–5.796)	
Alcohol		0.0688		0.3797
N	1.0		1.0	
Y	2.164 (0.942–4.969)		1.620 (0.552–4.750)	
Areca nut		0.3419		0.0365 *
N	1.0		1.0	
Y	0.699 (0.334–1.462)		0.413 (0.181–0.946)	
Smoking		0.2833		0.4950
N	1.0		1.0	
Y	1.913 (0.585–6.260)		1.678 (0.380–7.413)	

* Statistically significant (*p* < 0.05).

## Data Availability

The data presented in this study are available on request from the corresponding author.
